# *In Vitro* and *Ex Vivo* evidence that pharmacological induction of the hypoxia response pathway efficiently restricts measles and Nipah virus infections

**DOI:** 10.1080/22221751.2025.2563067

**Published:** 2025-09-17

**Authors:** Lola Canus, Clémence Jacquemin, Walid El Orch, Eva Ogire, Marion Ferren, Elodie Moissonnier, Alexandre Lalande, Amélie Bourgeais, Garance Ducret, Jules Bouget, Zofia Haftek, Florentine Jacolin, Anne Aublin-Gex, Didier Décimo, Marie Moroso, Stéphane Mely, Aurore Rozières, Mathias Faure, Olivier Diaz, Denis Gerlier, Mustapha Si-Tahar, Vincent Lotteau, Laure Perrin-Cocon, Cyrille Mathieu, Pierre-Olivier Vidalain

**Affiliations:** a CIRI, Centre International de Recherche en Infectiologie, Team NeuroInvasion, Tropism and Viral Encephalitis, Univ Lyon, Inserm, U1111, CNRS, UMR5308, Universite Claude Bernard Lyon 1, Ecole Normale Supérieure de Lyon, Lyon, France; b CIRI, Centre International de Recherche en Infectiologie, Team Viral Infection, Metabolism and Immunity, Univ Lyon, Inserm, U1111, CNRS, UMR5308, Universite Claude Bernard Lyon 1, Ecole Normale Supérieure de Lyon, Lyon, France; c Laboratoire P4 INSERM-Jean Mérieux, Lyon, France; d CIRI, Centre International de Recherche en Infectiologie, Team Autophagy Infection Immunity, Univ Lyon, Inserm, U1111, CNRS, UMR5308, Universite Claude Bernard Lyon 1, Ecole Normale Supérieure de Lyon, Lyon, France; e Inserm, U1100, Centre d'Etude des Pathologies Respiratoires (CEPR), University of Tours, Tours, France

**Keywords:** Nipah virus, measles virus, hypoxia-inducible factor, antiviral, Molidustat

## Abstract

Airborne RNA viruses of the *Paramyxoviridae* family are major human pathogens. These include measles virus (MeV) and Nipah virus (NiV), the latter being on the World Health Organization’s blueprint list due to its high case-fatality rate and critical risk of emergence. Although an effective vaccine is available for MeV, this is not the case for NiV. Moreover, there is no cure for MeV- or NiV-infected patients that prevents the acute respiratory syndrome or lethal encephalitis. To identify new host factors to target for inhibiting viral growth, a library of metabolic modulators was screened for activity against MeV using an *in vitro* infection model. Results showed that Molidustat, a pharmacological inhibitor of Prolyl-Hydroxylase Domain (PHD) enzymes, inhibits MeV infection in a Hypoxia-Inducible Factor (HIF)-dependent manner. We then tested the antiviral effect of Molidustat in organotypic cultures of hamster cerebellum. Molidustat induced the hypoxia-response pathway in this *ex vivo* model as assessed by transcriptomic analysis, and inhibited MeV infection. A similar antiviral effect was observed with Roxadustat and Daprodustat, two PHD enzyme inhibitors chemically unrelated to Molidustat. Finally, we showed that Molidustat inhibits NiV infection in organotypic cultures of hamster cerebellum and lung, thereby validating its effect in the two organs mainly targeted during infection. Taken together, our results provide evidence that pharmacological activation of the hypoxia-response pathway restricts MeV and NiV infections, highlighting HIF-inducing drugs as promising candidates to consider in the development of treatments.

## Introduction

*Paramyxoviridae* is a family of enveloped viruses with a negative, single-strand RNA genome. This family includes viruses which had a major impact on human health throughout history, such as measles virus (MeV). It is one of the most contagious viruses known and can be devastating in the absence of widespread vaccination. MeV causes acute systemic infections that are characterized by a cutaneous rash, fever, cough and general immunosuppression that predisposes patients to severe complications. In immunocompromised individuals, MeV can also penetrate the central nervous system (CNS), leading to MeV inclusion body encephalitis (MIBE) a few days to months after infection or, in rare cases, vaccination [1]. In a small proportion of immunocompetent patients, MeV can spread to the CNS years after the initial infection, resulting in subacute sclerosing panencephalitis (SSPE). To date, no treatment for these severe neurological complications is available.

Nipah virus (NiV) and its close relative Hendra virus (HeV) belong to the same viral family. They are highly pathogenic viruses that represent a major threat to humans. They are transmitted via respiratory droplets and cause outbreaks with a fatality rate in the 40–100% range. Although their natural reservoir lies in fruit bats (*Pteropus spp.*), both NiV and HeV can infect humans and numerous other mammals. For example, pigs and horses have been infected during outbreaks. For this reason, NiV and HeV present a very high risk of emergence [2]. There is no treatment or vaccine available for humans, and due to their fatality rate and human-to-human transmission capacity, they must be manipulated under biosafety level-4 laboratory (BSL4) conditions. NiV and HeV are on the World Health Organization (WHO) blueprint list of priority pathogens, which underscores the urgent need to develop effective countermeasures against them. Finally, NiV and HeV could be used as biological weapons, placing them in the category of dual-use agents. Since reverse genetics is now well established for them and MeV, they can be engineered for intentional dissemination.

Viruses can hijack the energy and metabolites of infected cells to fuel their replication and have evolved mechanisms to exploit host resources to match their needs [3]. This dependency makes cellular metabolism an attractive target for the development of Host-Directed Antivirals (HDAs) that aim to deprive the virus of resources and pathways it needs to grow [3]. To identify candidate host targets for HDAs against *Paramyxoviridae*, we screened a metabolism-oriented library of 493 drugs. Taking the advantage of the possibility to safely manipulate MeV in a biosafety level-2 laboratory (BSL2), we used it as a surrogate for NiV. Indeed, both MeV and NiV belong to the *Paramyxovirinae* sub-family and share many features of their genome organization and replication process. The screening led to the identification of pharmacological inducers of the hypoxia-response pathway as inhibitors of MeV. This drew our attention, as close interactions between viruses and this pathway have been reported previously [4]. The cellular response to hypoxia is controlled by Hypoxia-Inducible Factors (HIFs), which is a family of heterodimeric transcription factors composed of an alpha subunit (HIF-1α, HIF-2α or HIF-3α) interacting with a beta subunit (HIF-1β). The mechanisms underlying HIF-1 and HIF-2 activation are now well described [5]. Under normoxic conditions, Prolyl-Hydroxylase Domain enzymes (PHDs) hydroxylate the HIF-1/2α subunits, flagging them for recognition by the von Hippel-Lindau disease tumour suppressor protein (VHL). VHL then ubiquitinylates HIF-1/2α subunits, promoting their proteasomal degradation. In contrast, under hypoxic conditions, the decreased PHD activity leads to the stabilization of HIF-1/2α subunits. These subunits accumulate, translocate to the nucleus and heterodimerize with HIF-1β to transactivate target genes involved in cellular adaptation to hypoxia.

The improved understanding of these mechanisms allowed the development of pharmacological PHD inhibitors such as Daprodustat, Roxadustat and Molidustat, which are currently approved for the treatment of anemia caused by chronic kidney disease in humans (Daprodustat, Roxadustat) and cats (Molidustat) [5]. By inhibiting PHDs, the drugs of this new class prevent the hydroxylation and degradation of HIF-1/2α subunits in the presence of oxygen. This allows triggering erythropoietin synthesis and thus stimulates erythropoiesis. As HIFs modulate cellular metabolism, stress response and immune activation, as well as cell proliferation, survival and differentiation, HIF-inducing drugs may have broader therapeutic applications [5]. Here, we show that HIF-inducing molecules inhibit MeV and NiV replication in both *in vitro* and *ex vivo* models, suggesting that they may be useful in the design of antiviral therapies against *Paramyxovirinae*.

## Material and methods

### Cells and reagents

Huh7 (kindly provided by Dr. Marco Binder; Heidelberg University; Germany) and Vero E6 cells (CelluloNet; SFR Biosciences; France) were grown at 37°C and 5% CO_2_ in DMEM, high glucose, with GlutaMAX (Gibco; France) supplemented with 10% fetal calf serum (Biosera; France) with 100 U/mL of penicillin/streptomycin (Gibco; France). Molidustat was obtained from TargetMol, Roxadustat and Daprodustat from Selleckchem. Stock solutions were prepared at 10 mM in DMSO for storage at −80°C and used at indicated concentrations. IPPA17-A04 was provided by Dr. Yves L. Janin (Muséum National d’Histoire Naturelle; France).

### Viruses

The recombinant Moraten and Schwarz vaccine strains of MeV, expressing firefly luciferase (MeV-Luc) and EGFP (MeV-EGFP), respectively, have been previously described [6,7]. Both MeV-Luc and MeV-EGFP viruses were amplified on Vero cells at 32°C. The recombinant, hyperfusogenic MeV strain IC323-EGFP-F S103I N462S N465S was obtained by introducing mutations in the F gene of the MeV IC323-EGFP plasmid (kindly provided by Yanagi, Kyushu University, Fukuoka, Japan). This variant is able to disseminate in the absence of known receptors thanks to mutations introduced into the F protein [8]. It was propagated and titrated in Vero-hSLAM/hCD150 cells. Another hyperfusogenic variant, MeV IC323-EGFP-F Q96P, which carries a mutation located in F2 and was obtained in lab studies but never emerged in nature [9], was used in parallel to infect cerebellum cultures in order to confirm observations made with IC323-EGFP-F S103I N462S N465S. This mutant also expresses EGFP as reporter. The recombinant Nipah Malaysia virus (GenBank: AY029767) expressing EGFP from an additional transcription unit located between the N and P genes (rNiV-EGFP) was obtained by reverse genetics as previously described [10] and amplified on Vero E6 cells. NiV infections were carried out at the INSERM Jean Mérieux BSL4 laboratory in Lyon (France) following the highest international standards for biological safety. The mouse-adapted influenza A/Scotland/20/74 (IAV H3N2) strain (a kind gift of Pr. Sylvie van Der Werf’s team; Pasteur Institute, Paris, France) was amplified and titrated on Madin-Darby Canine Kidney (MDCK) cells.

### Screening of the chemical library

The Metabolism-related Compound Library of APExBio (493 compounds; Ref. L1032; DiscoveryProbe; APExBio Technology) was purchased from Stratech. Unless specified otherwise by the manufacturer, drugs were provided as stock solutions at 10 mM in DMSO and stored at −80°C. The screen was carried out in white flat-bottom 96-well screening plates (Greiner) containing 1 × 10^4^ Huh7 cells and 200 μL of culture medium in each well. Each drug was screened individually in a single well at a final concentration of 50 μM. Cells were infected with MeV-Luc on the same day or the day after at a multiplicity of infection (MOI) of 0.5. The viral inoculum was adjusted to account for the approximate doubling of the cell population in 24 h. Each screening plate contained 4 negative control wells treated with DMSO alone and 4 positive control wells treated with IPPA17-A04 at 2 μM [11]. After 48 h of culture in the presence of drugs, 100 μL of medium was removed from each well, and luciferase activity was quantified by adding 50 μL of Bright-Glo Luciferase assay reagent (Promega). Luminescence was measured after 5 min of incubation at room temperature with a Tristar luminometer from Berthold. Statistical parameters of the screening, including Z’ factors, signal/background and signal/noise ratios, were calculated as previously described [12]. Drug toxicity was assessed in parallel by growing Huh7 cells with compounds of the library at 50 μM. After 3 days of culture, ATP levels in culture wells were quantified with the CellTiter-Glo Luminescent Cell Viability reagent (Promega), and results were expressed as percentage of the values from untreated control wells. Drugs reducing the luminescence signal by more than 40% were considered toxic and discarded.

### Quantification of secreted CXCL10, IFN-λ1 and IL-6

Huh7 cells were infected with MeV-Luc (MOI = 0.4 or 1.6) and treated with Molidustat. After 48 h of culture, supernatants were collected, and the LEGENDplex Human Anti-Virus Response Panel (13-plex; BioLegend) was used to quantify a panel of 13 cytokines. Only CXCL10, IFN-λ1 and IL-6 were strongly induced by MeV infection and quantified.

### Measuring MeV-EGFP growth in Huh7 cells

Huh7 cells were plated in 24-well plates at 5 × 10^4^ cells per well. After 24 h of culture, cell culture medium was removed and replaced by 500 μL of fresh medium containing DMSO or Molidustat at 12, 25 or 50 μM. After 24 h of incubation, MeV-EGFP was added at a MOI of 1 (it was estimated that each well contained 1.4 × 10^5^ cells at the infection timepoint). At 24 h post-infection, cells were washed with PBS and fixed with PFA 4% at 4°C for at least 1 h. PFA was removed and cells were permeabilized for 15 min at room temperature with 0.5% Triton in PBS. Cells were washed twice with PBS and nuclei were stained with Hoechst at 1.5 μM in PBS for 30 min at 37°C. Cells were washed twice with PBS and fluorescence in the green (λex. 483 nm/λem. 536 nm) and blue (λex. 377 nm/λem. 447 nm) channels was analyzed on a Celigo Image Cytometer (Revvity). Image mosaics were reconstituted by stitching using Celigo software. Images were analyzed with ImageJ to quantify overall Hoechst and EGFP signal intensities after background subtraction (set to 30 for Hoechst and 45 for EGFP) and image thresholding (range set to 20–255 for both channels).

### Knock-down by siRNA transfection in Huh7 cells

The siRNAs targeting HIF-1α (L-004018-00), HIF-2α (L-004814-00), and the Non-targeting Control Pool siRNA (D-001810-10) were obtained from Horizon Discovery in the ON-TARGETplus SMARTpool format. The siRNA targeting ATG5 (HSS190366) and the Stealth RNAi™ siRNA Negative Control (12935300) were purchased from Life Technologies.

Huh7 cells were seeded in 96-well plates at 7 × 10^3^ cells per well in 100 µL of culture medium. After 24 h of incubation, the supernatant was removed and replaced with fresh medium without antibiotics. Cells were transfected with 0.5 µL of each siRNA at 5 µM per well using DharmaFECT Transfection Reagent (Horizon Discovery), following the manufacturer’s guidelines. After 6 h of incubation, cells were treated with Molidustat (25 μM) or DMSO alone. After 24 h of culture, cells were infected with MeV-Luc (MOI = 0.5). After an additional 24 h of culture, cells were harvested and processed using the Bright-Glo Luciferase Assay System (Promega) to quantify MeV-Luc expression.

Validation of HIF-1/2 knockdown has been previously reported [13]. To determine ATG5 knockdown by qPCR, cells were washed with PBS and processed using the Direct-zol RNA Miniprep Kits (Zymo Research). Reverse transcription was performed using the High-Capacity RNA-to-cDNA Kit (Applied Biosystems) following the manufacturer’s recommendations. qPCR was carried out using the PowerTrack SYBR Green Master Mix (Applied Biosystems) on a CFX96 Real-Time PCR Detection System (Bio-Rad). ATG5 primers were designed with Primer-BLAST and human RPL13A (hRPL13A) were from Xiao J et al. [14] (Table 1). Gene expression was analyzed as fold change using the 2^−ΔΔCt^ method [15], with hRPL13A as the housekeeping gene. Decreased ATG5 protein expression was determined by western-blot. Protein extracts were separated on a Mini-PROTEAN TGX Precast Protein Gel 4–20% (Bio-Rad; Ref: 4561094). After transfer on a nitrocellulose membrane (Trans-Blot Turbo Mini 0.2 µm Nitrocellulose Transfer Packs; Bio-Rad), ATG5 was detected with a rabbit polyclonal antibody (Cell Signaling Technology; Ref: 2630) diluted 1000 times in 1X TBS buffer (Euromedex; Ref: ET220-B) containing 0.1% Tween and 5% fat-free milk. Cyclophilin B (CYCP), used as a loading control, was detected with a rabbit monoclonal antibody (Cell Signaling Technology; Ref: 43603) under the same conditions. The HRP-conjugated secondary antibody was from Sigma-Aldrich (Ref: A0545). The molecular weight marker was from Euromedex (Ref: 06P-0211). The luminescent signal was quantified with the ChemiDoc Imaging System (Bio-Rad).

**Table 1.** List of primers used for RT-qPCR.

Name	Sequence
MeV-N_For	5′ GTGATCAAAGTGAGAATGAGCT 3′
MeV-N_Rev	5′ GCTGACCTTCGACTGTCCT 3′
NiV-N_For	5′ GGCAGGATTCTTCGCAACCATC 3’
NiV-N Rev	5′ GGCTCTTGGGCCAATTTCTCTG 3’
Hamster RPL13A For	5′ GTAACGGCCACACTGGAAGA 3’
Hamster RPL13A Rev	5′ TGTTTCCGTAGCCTCACCAG 3’
NDUFA4L2 For	5′ ATGGCAGGAACCAGTGCAG 3’
NDUFA4L2 Rev	5′ CCGGTCCTTCTTCAGCTTCT 3’
BNIP3 For	5′ CCCAAGCGCACAACTACTCT 3’
BNIP3 Rev	5′ AGGTGCTGGTGGAAGTTGTC 3’
BNIP3L For	5′ AGACCCGAAAACATCCCACC 3’
BNIP3L Rev	5′ CAGAAGGTGTGCTCAGTCGT 3’
MX1 For	5’ CTTCAAGGAGCACCCACACT 3’
MX1 Rev	5’ CTTGCCCTCTGGTGACTCTC 3’
CXCL10 For	5’ AGACAACAGTAACTCCAGTGACAAG 3’
CXCL10 Rev	5’ AGTGTAGCACCTCAGCGTAGC 3’
hATG5 For	5′ GCTGTTTCGTCCTGTGGCTG 3’
hATG5 Rev	5′ TTCAATCTGTTGGCTGTGGGA 3’
Human RPL13A For	5′ AAAAGCGGATGGTGGTTCCT 3’
Human RPL13A Rev	5′ GCTGTCACTGCCTGGTACTT 3’
IAV-M For	5’ AAGACCAATCCTGTCACCTCTGA 3’
IAV-M_Rev	5’ CAAAGCGTCTACGCTGCAGTCC 3’
Murine Rplp0 For	5’ GGCATCACCACGAAAATCTCC 3’
Murine Rplp0 Rev	5’ GACACCCTCCAGAAAGCGAG 3’

### Animals and ethical authorization

Suckling Syrian Golden hamsters (*Mesocricetus auratus*) and suckling C57BL/6 mice used in our study were obtained from Janvier Labs (Le Genest-Saint-Isle, France) with clean health monitoring reports. The sex of the animals was random and dependent on the litter birthed by the mother. All animals were used and euthanized by decapitation at 7 days of age according to recommendations of the American Association for Accreditation of Laboratory Animal Care (AAALAC) and according to French Ethical Committee (CECCAPP) regulations, accreditation # CECCAPP_ENS_2014_034. *Ex vivo* models have been developed specially to follow the 3R’s rule (Replacement, Reduction, Refinement).

### Preparation of organotypic cerebellum and lung cultures

Organotypic Cerebellum Cultures (OCCs) and Organotypic Lung Cultures (OLCs) were prepared from 7-day-old Syrian golden hamsters and cultured as detailed elsewhere [16,17]. Briefly, the day before dissection, Millicell cell culture inserts (30 mm, hydrophilic polytetrafluoroethylene, Merck Millipore) were incubated overnight in organotypic culture medium. This medium consists of Minimal Essential Medium GlutaMAX (Thermo Fisher Scientific) supplemented with 25% heat-inactivated horse serum (Gibco), D-glucose (5 g/L; Sigma-Aldrich) and human recombinant insulin (0.02 mg/mL; Sigma-Aldrich), and was sterilized with a 0.22 μm filter. Hamster cerebella and lungs were isolated by dissection and sliced with a McIlwain tissue chopper (WPI-Europe) to generate 350 and 400 µm-thick explants, respectively. Slices were then collected in a solution of Hibernate®-A medium (Sigma-Aldrich) containing 1X penicillin/streptomycin solution (Gibco), and supplemented with 0.189 mg/mL kynurenic acid (Sigma-Aldrich) for OCCs, transferred and cultured on insert with organotypic culture medium at 37°C in 5% CO_2_. OCCs were infected on the day of slicing with 10,000 plaque-forming units (PFUs) per slice of MeV IC323-EGFP-F S103I N462S N465S or Q96P, or with 200 PFUs per slice of rNiV-EGFP. 1 h after infection, the slices were treated with DMSO, Molidustat, Daprodustat or Roxadustat by diluting the drugs in the culture medium at 25 µM and then pipetting 3 µL drops on the slices. Slices were maintained in culture at 37°C in 5% CO_2_ for 48 and 72 h without daily refills, since these drugs are known for being stable for several hours *in vivo*. For murine OLCs (mOLCs), lungs from 7-day-old C57BL/6 mice were prepared as hamster OLCs and maintained in Hibernate®-A medium with 1% P/S without kynurenic acid. On the day of the slicing, mOLCs were infected with 2,000 PFU of IAV H3N2 and then cultured with either DMSO alone or Molidustat (25 µM) as described above.

### Slice imaging

OCCs infected with MeV were imaged using a Nikon Eclipse Ts2R optical microscope at 200 ms exposure for DMSO or Molidustat, and 300 ms exposure for DMSO or Roxadustat with a 4X objective. Slices infected with rNiV-EGFP were imaged with a Leica DMIRB microscope at 100 ms exposure with a 5X objective. Images were stitched and analyzed using the ImageJ software 1.52p FiJi package (stitching plugin) and assembled in Inkscape.

### Viability test on organotypic brain cerebellum cultures

The viability of OCCs treated either with DMSO, Molidustat, Roxadustat or Daprodustat at 25 µM was assessed using the MTT Cell Viability Assay (Thermo Fischer Scientific) after 72 h of treatment. 70 µl of MTT solution was added on top of each slice. Slices were incubated for 4 h at 37°C. Each slice was transferred into a well of a 48-well plate and covered with 200 µl of DMSO to solubilize formazan. After 10 min of incubation at 37°C, 75 µl of DMSO containing formazan were transferred to a 96-well plate for absorbance measurement at 540 nm (TECAN M200).

### Real-Time quantitative PCR in tissues

OCCs infected with MeV IC323-EGFP-F were collected in 350 µL of TRI reagent (Euromedex) with two stainless steel beads (Next Advance) and lysed using a TissueLyser II (Qiagen) at Vmax (30 Hz) for 10 min. OCCs infected with NiV were collected in 1 mL of TRIzol (Thermo Fischer Scientific) and lysed using the same protocol. RNA was extracted using Direct-zol RNA Miniprep Kits (Zymo Research). 200 ng of RNA was reverse transcribed with the iScript cDNA Synthesis Kit (Bio-Rad), and cDNA was diluted 1:10. Quantitative PCR was performed using the Platinum SYBR Green qPCR SuperMix-UDG (Invitrogen) on a StepOnePlus Real-Time PCR System (Applied Biosystems). Primer pairs were designed with Beacon Designer (version 8) software and selected for an efficacy close to 100% according to the MIQE checklist [18]. Hamster RPL13A used as housekeeping gene was quantified using previously described primers [19]. All samples were run in duplicates and results were analyzed using StepOne version 2.3 software (Applied Biosystems). All calculations were done according to the 2^-ΔΔCT^ model [15]. Calculations of the copy numbers were normalized to the standard deviation (SD) of the housekeeping gene (RPL13A) mRNA as formerly described [17].

For mOLCs, lung slices were lysed in 350 µL of RA1 lysis buffer (Macherey Nagel) + 1% β-mercaptoethanol in Precellys tubes with two steel beads (Next Advance) using the following protocol: 3x (15s 6500 rpm – 15s break) with the Precellys Evolution lyser (Bertin). RNA was isolated using RNA NucleoSpin kit (Macherey Nagel) according to the manufacturer’s instructions. 350 ng of RNA were transcribed into cDNA using the High-Capacity RNA-to-cDNA kit (Applied Biosystems), and quantitative PCR was carried out on 10-fold diluted cDNA templates with 10 µM of specific primers with Quantinova® SYBR® Green PCR kit (Qiagen) on a StepOnePlus Real-Time PCR System (Applied Biosystems). IAV primers were from Guillon A. et al [20] (Table 1). Murine Rplp0 was used as housekeeping gene and was quantified using primers designed with Primer-BLAST (Table 1).

### Transcriptomic analysis

Cerebellum slices from different individuals were incubated as described above on inserts for 24 h in presence of DMSO alone (n = 4) or Molidustat at 25 µM (n = 5). OCCs treated either with DMSO or Molidustat were collected in 350 µL of TRI reagent (Euromedex) with two stainless steel beads and lysed using a TissueLyser II (Qiagen) at Vmax (30 Hz) for 10 min. Next, RNA was extracted using Direct-zol RNA Miniprep Kits (Zymo Research). DNA library construction, sequencing and data analysis described here were performed by Microsynth AG (Balgach, Switzerland). Total RNA was quantified using Quant-iT RiboGreen (Thermo Fisher Scientific) and integrity was checked on Fragment analyzer (Agilent) with a kit suitable for RNA. Library Preparation was done using the Illumina® Stranded mRNA Prep, Ligation and 60 ng total RNA input. Final libraries were quantified using pico488 (Lumiprobe) and library size was checked on Fragment analyzer (Agilent). Samples were pooled equimolar prior to sequencing. Subsequently, the Illumina NovaSeq platform and an SP 300 cycles kit were used to sequence the libraries in a paired-end fashion. The produced paired-end reads, which passed Illumina’s chastity filter, were subject to de-multiplexing and trimming of Illumina adaptor residuals using Illumina’s bcl2fastq software version 2.20.0.422 (no further refinement or selection). Quality of the reads in fastq format was checked with the software FastQC (version 0.11.8; https://www.bioinformatics.babraham.ac.uk/projects/fastqc/). Raw reads having average Q-values below 24 or incorporating uncalled “N” bases or being smaller than 25 bases after trimming were filtered using the BBTools software suite (version 38.86; https://sourceforge.net/projects/bbmap/). The splice aware RNA mapping software STAR (version 2.7.10a) [21] was used to map the surviving reads to the GCF_017639785.1 reference genome provided by NCBI. To count the uniquely mapped reads to annotated genes, the software htseq-count (HTSeq version 0.13.5) was used [22]. Normalization of the raw counts and differential gene expression analysis was carried out with help of the R software package DESeq2 (version 1.26.0) [23]. Raw data are available on the Gene Expression Omnibus database (Accession number GSE294415). The functional enrichment analysis was limited to well-annotated, upregulated genes, and was performed with DAVID using KEGG annotation for *Mus musculus* [24–26]. The Gene Set Enrichment Analysis (GSEA) was performed with WebGestalt using MSigDB hallmark gene sets for “Hypoxia,” “Inflammatory response” and “Interferon alpha response” [27,28].

### Statistical analysis and other software

**Figure 1.**
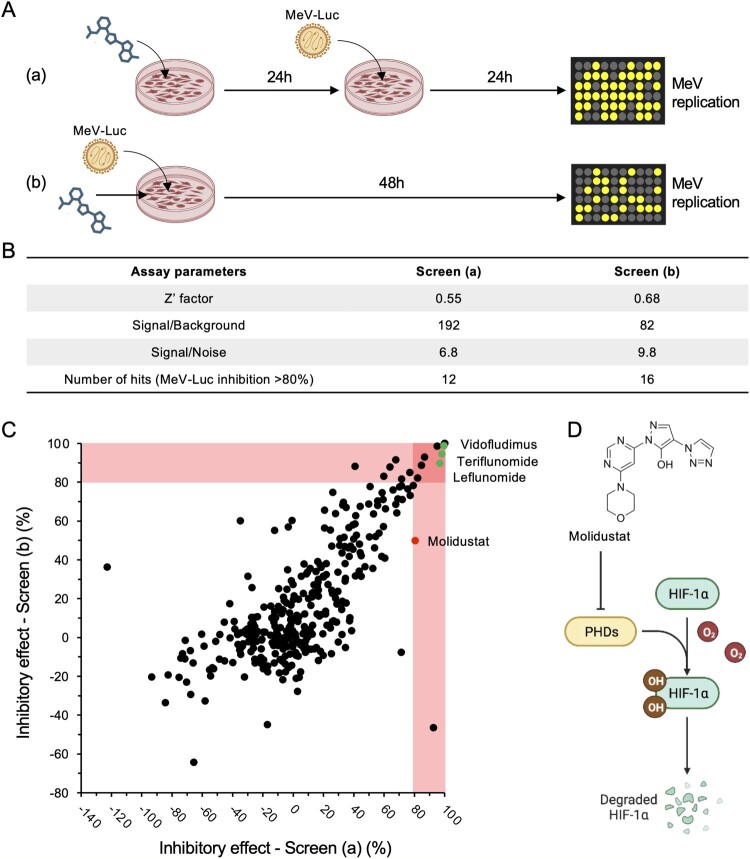
Identification of MeV inhibitors by screening a chemical library of metabolic modulators. (A) A total of 493 compounds were tested at 50 μM on Huh7 cells infected with MeV-Luc (MOI = 0.5). Drugs were added either 24 h before infection (a) or at the time of infection (b). Luciferase activity was determined at 24 h (a) or 48 h (b) post-infection, and results were normalized across plates using DMSO-treated control wells as a reference. (B) Statistical parameters of the two screening protocols. Hits correspond to drugs inhibiting MeV-Luc infection by more than 80% without affecting cell count or viability (see M&M for details). (C) Dot plot showing the inhibitory effect of the drugs according to screening protocols (a) and (b). Drugs considered toxic (see M&M for details) are not displayed. (D) Chemical structure of Molidustat and schematic showing the hypoxia response pathway.

All statistical analyses were performed with GraphPad Prism v10. EC_50_ and EC_90_ values were determined by nonlinear regression fitting using a four parameters model. Figure 1A, 1D and Figure 3A were created in BioRender. Vidalain, P. (2025) https://BioRender.com/f96b399 and Mathieu, C. (2025) https://BioRender.com/v34z384.

## Results

### Screening a library of metabolism-oriented compounds to identify MeV inhibitors

To identify cellular metabolic factors that could be targeted with drugs to inhibit infection by *Paramyxoviridae*, we screened a metabolism-oriented library of 493 compounds *in vitro*. Our assay was based on human cells infected with a recombinant vaccine strain of luciferase-expressing MeV (MeV-Luc), which allowed us to monitor the viral infection progression. As previously reported, this provides a robust platform for screening chemical libraries in high-throughput setting [29]. Although hepatocytes are not a natural target of wild-type MeV, we used Huh7 hepatocellular carcinoma cells due to their high permissiveness to the MeV vaccine strain and their well-characterized and broad metabolic functionality [13]. Two screens were carried out in parallel. In the first one, the drugs and the virus were administered simultaneously to the cells. In the second one, the cells were pre-treated with the drugs 24 h before infection (Figure 1A). As a positive control, we used IPPA17-A04, a well-described inhibitor of *de novo* pyrimidine biosynthesis that targets dihydroorotate dehydrogenase (DHODH) and exhibits broad-spectrum antiviral properties [11]. DMSO was used as a negative control. Z’ values above 0.5 confirmed the reliability of the assay (Figure 1B), thus validating our screening procedure [12]. To minimize cytotoxic and cytostatic bias, we first excluded the 161 compounds that reduced ATP levels by more than 40% after 72 h in Huh7 cells (measured by CellTiter-Glo), thus remaining with 332 non-cytotoxic candidates. Among these, 18 compounds inhibited MeV-Luc infection by over 80% (Figure 1C). As expected, three were DHODH inhibitors (Leflunomide, Teriflunomide, and Vidofludimus), further supporting the relevance of metabolic targeting in antiviral strategies. Of particular interest was Molidustat, a Prolyl-Hydroxylase Domain (PHD) inhibitor that has been developed to treat anemic patients by stimulating the HIF pathway (Figure 1D) [5]. Interestingly, its antiviral activity was more pronounced when cells were pretreated before infection, suggesting that HIF pathway activation may induce a cellular state that interferes with MeV replication. We thus decided to further investigate this antiviral effect.

### MeV infection is restricted by PHD inhibitors via HIF1/2α

**Figure 2.**
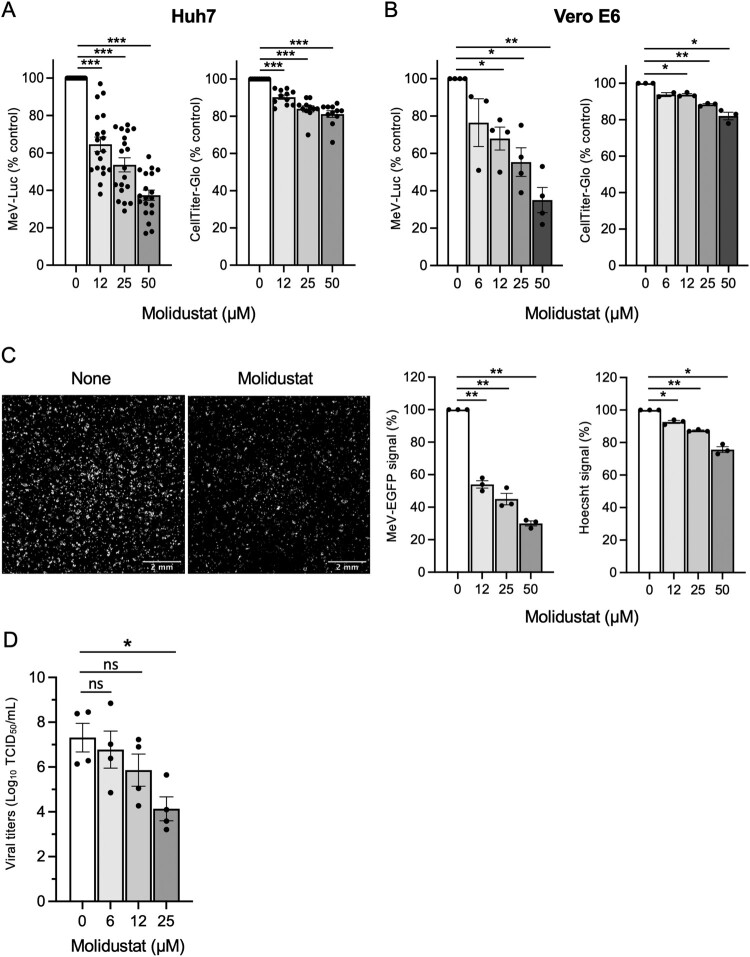
Molidustat inhibits MeV replication in Huh7 and Vero cell lines. (A) Huh7 cells were treated with Molidustat at indicated concentrations for 24 h and then infected with MeV-Luc (MOI = 0.5). After an additional 24 h of culture in the presence of Molidustat, luciferase activity was determined and results were normalized using DMSO-treated control wells as a reference (left panel). In parallel, Huh7 cells were treated with Molidustat for 48 h and the number of viable cells was evaluated using the CellTiter-Glo reagent (right panel). (B) Same experimental setting as in (A) performed on Vero cells. (A-B) Each dot represents the mean value of one experiment performed in triplicate. (C) Huh7 cells were treated with Molidustat at indicated concentrations for 24 h and then infected with MeV-EGFP (MOI = 1). After an additional 24 h of culture in the presence of Molidustat, culture wells were imaged by fluorescence microscopy to quantify EGFP expression. Left panel: representative images of EGFP expression in infected cultures that were treated with DMSO (None) or Molidustat at 25 µM. Middle and Right panels: viral growth and cell density were determined by image analysis based on EGFP expression and nuclei staining with Hoechst, respectively. Results were expressed as percentage of the overall EGFP and Hoechst signals normalized to DMSO-treated control wells. Each dot represents the mean value of one experiment performed in duplicate. (A-C) Mean ± SEM; *p < 0.05, **p < 0.01, ***p < 0.001; One sample t-test using 100% as a reference and Holm-Šidák correction for multiple testing. (D) Huh7 cells were treated with Molidustat at the indicated concentrations for 24 h and then infected with MeV-Luc (MOI = 0.05). After an additional 48 h of culture in the presence of Molidustat, cultures were stopped by freezing at −20°C. After thawing the samples, the production of infectious particles was determined by titration on Vero E6 cells using the TCID_50_ method. Each dot represents the mean value of one experiment performed in quadruplicate. Mean ± SEM; *p < 0.05; Paired Student’s t-test and Holm-Šidák correction for multiple testing.

To validate the effect of Molidustat on MeV infection, Huh7 cells were pretreated for 24 h with increasing concentrations of the drug prior to infection with MeV-Luc. Quantification of the luciferase signal 24 h after infection confirmed the inhibitory effect of Molidustat. Its half maximal effective concentration (EC_50_) was estimated to 27 μM (Figure 2A). Consistent with our recent report [13], 48 h of Molidustat treatment had a limited impact on Huh7 cell proliferation and viability as assessed by ATP quantification in culture wells. Molidustat exhibited a similar antiviral effect in Vero E6 cells (EC_50_ = 28 μM; Figure 2B). To replicate these findings using a different readout system, Huh7 cells were treated with Molidustat for 24 h and then infected with a recombinant strain of MeV expressing EGFP. After 24 h, cell layers were imaged by fluorescence microscopy to quantify overall EGFP expression (Figure 2C; left panel). The results confirmed a dose-dependent inhibition of viral infection by Molidustat (Figure 2C; middle panel; EC_50_ = 21 μM). Importantly, we showed that Molidustat had a limited impact on cell counts as assessed by DNA labelling with Hoechst staining (Figure 2C; right panel). Finally, we measured the effect of Molidustat on the production of infectious particles when Huh7 cells were pretreated for 24 h with Molidustat before infection with MeV-Luc. Infectious virus particles were quantified in infected cultures by titration using the TCID_50_ method. Results showed that Molidustat effectively reduced the production of infectious virions by 3 logs at 25 μM (Figure 2D), and the calculation estimated the EC_90_ to be around 10 μM.

**Figure 3.**
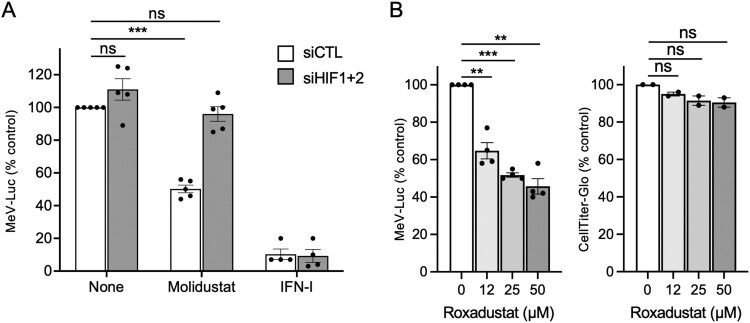
MeV infection Huh7 cells is inhibited by activation of the HIF pathway. (A) Huh7 cells were transfected with either a mix of siRNA targeting both HIF-1α and HIF2α or a control siRNA. After 6 h of culture, cells were treated with DMSO (None), Molidustat at 25 µM or recombinant IFN-α (100 IU/mL). After 24 h of culture, cells were infected with MeV-Luc (MOI = 0.5). After an additional 24 h of culture in the presence of Molidustat or IFN-α, luciferase activity was determined and results were normalized using DMSO-treated control wells as a reference. (B) Huh7 cells were treated with Roxadustat at indicated concentrations for 24 h and then infected with MeV-Luc (MOI = 0.5). After an additional 24 h of culture, luciferase activity was determined and results were normalized using DMSO-treated control wells as a reference (left panel). In parallel, Huh7 cells were treated with Roxadustat for 48 h and the number of viable cells was evaluated using the CellTiter-Glo reagent (right panel). Mean ± SEM; *p < 0.05, **p < 0.01, ***p < 0.001; One sample t-test using 100% as a reference and Holm-Šidák correction for multiple testing.

We previously demonstrated that Molidustat increases the expression of both HIF-1α and HIF-2α at the protein levels [13]. To study the role of these transcription factors in the antiviral mechanism of action of Molidustat, HIF-1α and HIF-2α were silenced by siRNA transfection following a previously validated protocol [13]. As shown in Figure 3A, the knock-down of HIF-1α and HIF-2α abrogated the inhibition of MeV-Luc infection by Molidustat. In contrast, inhibition of MeV-Luc by type I interferon (IFN-I) was not affected. To confirm that the observed antiviral activity was due to HIF pathway activation rather than an off-target effect of Molidustat, we tested Roxadustat, a PHD inhibitor which is chemically unrelated to Molidustat. Roxadustat also inhibited MeV-Luc infection in Huh7 cells (EC_50_ = 34 μM; Figure 3B). Overall, these results demonstrate that the activation of the HIF pathway via PHD inhibition restricts MeV infection *in vitro*.

The HIF pathway can regulate the innate immune response, but these interactions are complex and opposing effects have been reported depending on the system analyzed. We therefore investigated whether Molidustat could induce interferon or inflammatory factors in Huh7 cells, which could explain its antiviral effect. To address this question, we reanalyzed transcriptomic data we previously obtained from Huh7 cells treated or not with Molidustat for 24 h (GSE242340) [13], for Gene Set Enrichment Analysis (GSEA) using MSigDB hallmark gene sets for the “Inflammatory” response and type I interferon response (labelled “Interferon alpha” in MSigDB) [27,28]. We previously established the strong induction of hallmark genes of the “Hypoxia” response in Molidustat-treated Huh7 cells [13]. In contrast, hallmark genes of the “inflammatory” and “interferon alpha” response sets were not significantly induced or repressed (Supplementary Fig. 1A; FDR>0.05). Furthermore, when 26.1% of Molidustat-induced genes (FC > 2; *p*-value<0.05) mapped to the “Hypoxia” response gene set, only 4.5% and 1.5% mapped to the “Inflammatory” and “Interferon alpha” gene sets, respectively (Supplementary Fig. 2A and B). In addition, we also evaluated the effect of Molidustat on the specific induction of CXCL10, IFN-λ1 and IL-6 by MeV infection (Supplementary Fig. 3). Molidustat did not increase but rather reduced their expression. This may be a direct effect of Molidustat or an indirect consequence of viral growth inhibition. In any case, it is therefore unlikely that the induction of interferon-stimulated genes or inflammatory factors is responsible for the antiviral effect of PHD inhibitors in Huh7 cells.

As autophagy contributes to the inhibition of Japanese Encephalitis Virus (JEV) and Herpes Simplex Virus 2 (HSV-2) by HIF-inducing drugs [30,31], we investigated whether canonical autophagy also contributed to MeV inhibition by Molidustat. Since ATG5 is a core autophagy factor and has been implicated in HSV-2 inhibition under hypoxia [31], Huh7 cells were transfected with ATG5-targeting siRNA or control siRNA as previously described [32], then treated with Molidustat 24 h before MeV-Luc infection. We observed that ATG5 silencing, validated by RT-qPCR and western-blot (Supplementary Fig. 4A-B), had no impact on the antiviral effect of Molidustat (Supplementary Fig. 4C-D), indicating that ATG5 was not required for MeV inhibition.

### HIF-inducing drugs inhibit MeV infection ex vivo

To overcome the limitations inherent to immortalized cell lines, we used an *ex vivo* infection model based on hamster cerebellum slices maintained in culture at an air–liquid interface [17]. Given the neurotropism of MeV and NiV, these organotypic cerebellum cultures (OCCs) have been established as a relevant *ex vivo* model to evaluate infection of the brain tissue by these viruses [17]. In addition, both viruses target this substructure which is one of the most essential for host survival. OCCs contain the full diversity of neural cells (including neurons, oligodendrocytes, astrocytes and microglia) and preserve the tridimensional architecture of the original tissue for several days. OCCs can be infected with a recombinant strain of MeV with F protein mutations found in multiple MIBE/SSPE virus isolates, reflecting virus cell tropism and fully support virus cell-to-cell dissemination [8,17]. These mutations enable the virus to fuse with host cell membranes in the absence of known high-affinity receptors. Hence, OCCs infected with this hyperfusogenic variant are a physiologically relevant model for testing antiviral strategies against MeV brain infection [17].

**Figure 4.**
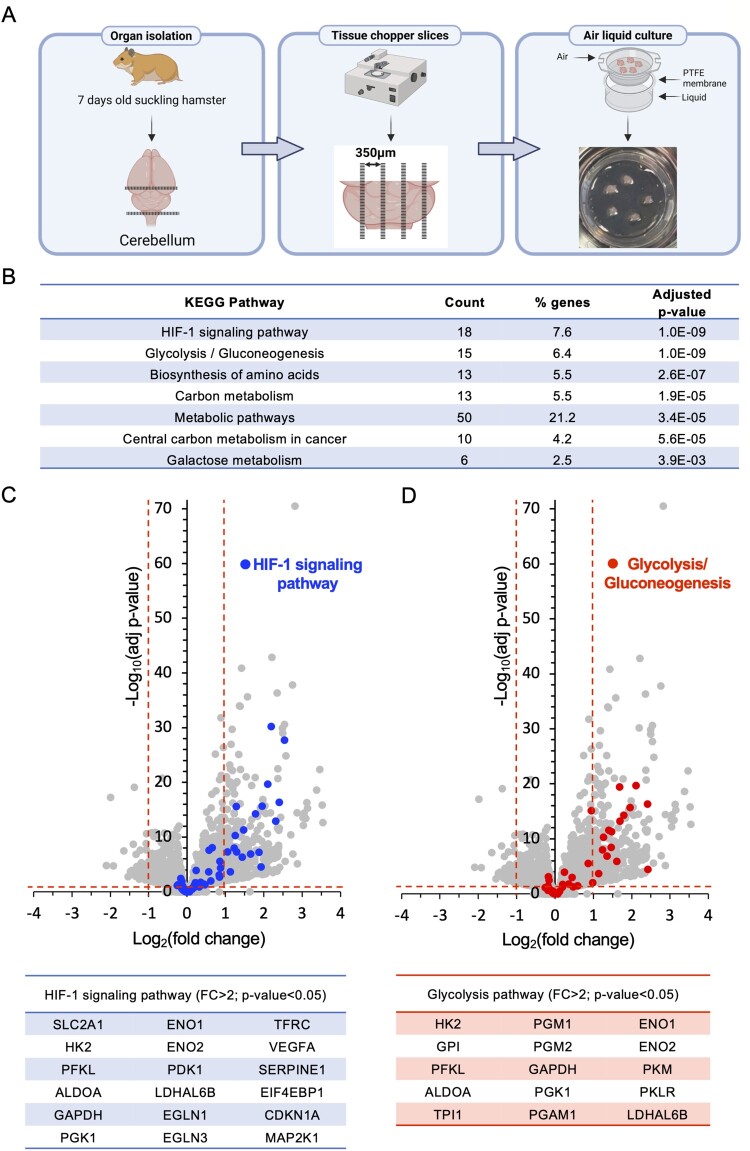
Molidustat activates the hypoxia response pathway in hamster organotypic brain cultures. (A) Schematic illustrating the procedure used to prepare hamster organotypic cerebellum cultures (OCCs). (B) OCCs were treated for 24 h with DMSO alone (n = 4) or Molidustat at 25 µM (n = 5). Tissue slices were collected and total RNA was extracted for transcriptomic analysis by NGS. Functional enrichment analysis of the 237 upregulated genes well-annotated in the hamster genome (FC > 2; adjusted *p*-value<0.05) using the KEGG database (annotation for *Mesocricetus auratus*). (C-D) Volcano plots showing regulated genes with a specific highlight on genes associated with the “HIF-1 signaling pathway” (C) or the “Glycolysis/Gluconeogenesis” (D) in the KEGG database. Names of upregulated genes are listed below each volcano plot.

The procedure for the preparation and culture of OCCs is illustrated in Figure 4A. Before testing the antiviral effect of Molidustat in this model, we first verified that hamster OCCs could respond to the drug. Slices were left untreated or treated with Molidustat at a concentration shown to be non-toxic in cell cultures (25 μM) [13], and their transcriptomes were analyzed by next-generation sequencing (NGS). Molidustat treatment inhibited the expression of 41 genes and induced 360 genes (Supplementary Table 1). KEGG pathway enrichment analysis of the 237 upregulated genes that are well annotated in the hamster genome revealed a significant induction of genes associated with the “HIF-1 signaling pathway” (Figure 4B). A volcano plot highlighting the 18 genes related to hypoxia in the KEGG database is shown, with their name listed in the table below (Figure 4C). Genes encoding enzymes of the glycolytic pathway were also induced, which is typical of cellular metabolic reprogramming upon the activation of the hypoxia-response pathway (Figure 4B and D) [13]. GSEA revealed similar gene enrichment in the MSigDB hallmark hypoxia response gene set (Supplementary Fig. 1B), with 13.9% of the 237 genes induced by Molidustat being associated with hypoxia (Supplementary Fig. 2A), including several glycolysis enzymes (Supplementary 2B). Manual curation of the dataset also identified the upregulation of canonical genes of the hypoxia-response pathway that, however, are not in the KEGG or MSigDB gene signatures. This includes NDUFA4L2 and BNIP3, which are involved in mitochondrial respiration inhibition and mitophagy, respectively [33,34]. Overall, this demonstrates that Molidustat activates the hypoxia response pathway in OCCs.

**Figure 5.**
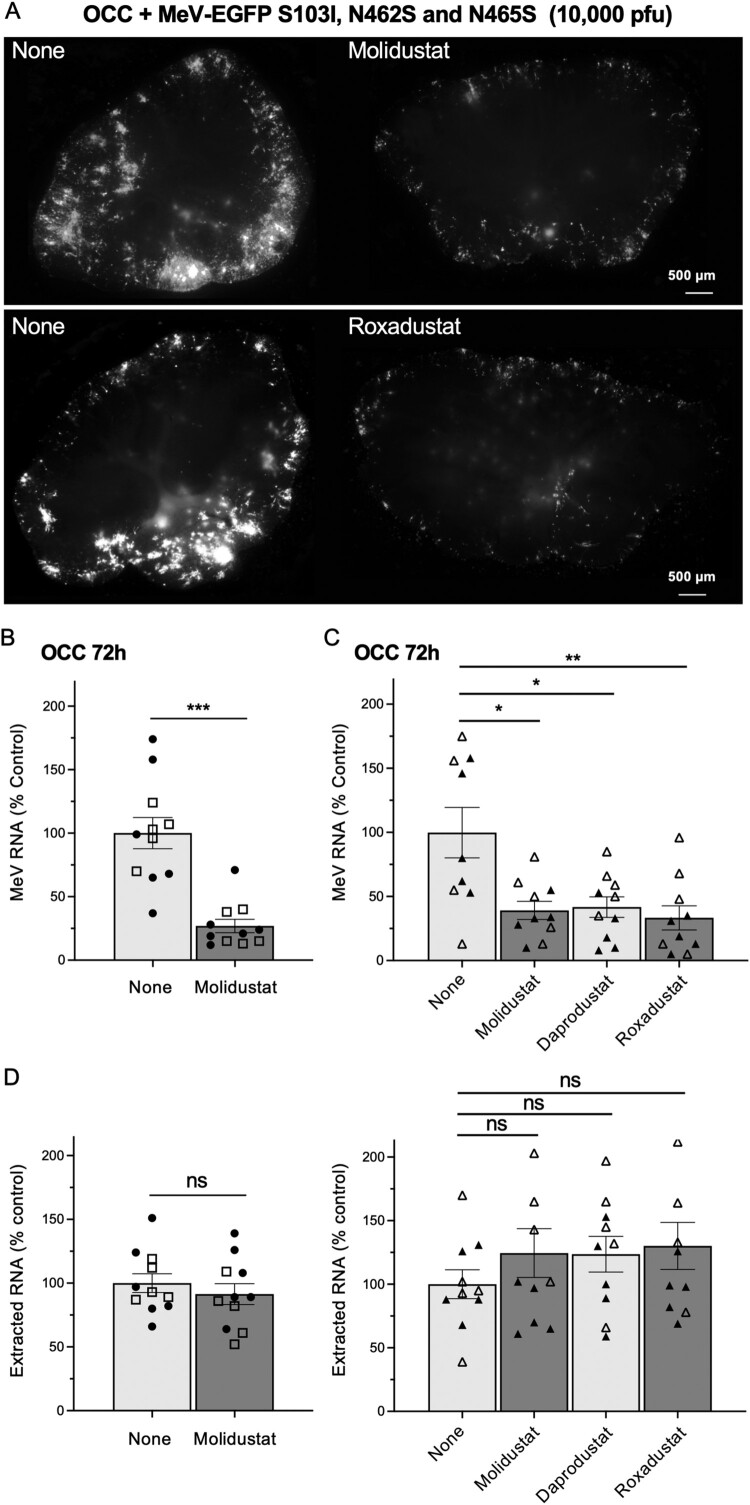
Pharmacological induction of the HIF pathway inhibits MeV replication in hamster organotypic brain cultures. Hamster OCCs were infected with hyperfusogenic MeV-EGFP (carrying S103I, N462S and N465S mutations in the F protein; 10,000 PFU/slice). One hour after infection, the indicated HIF-inducing drugs were added at 25 µM and MeV infection was analyzed after 72 h of culture. (A) Fluorescence images of infected OCCs that were either left untreated (DMSO) or treated with Molidustat or Roxadustat. (B) Quantification of MeV infection by RT-qPCR. Each dot represents one tissue slice from one animal. The whole experiment has been repeated twice, and replicates are identified by open squares and closed circles. For each tissue slice, MeV-N RNA levels were first normalized to the RPL13A mRNA level, then to the average signal obtained across all DMSO-treated slices of the corresponding experiment. (C) The presented data correspond to a set of two experiments with the same setup as in (B) identified by open and closed triangles. Roxadustat and Daprodustat were included in parallel to Molidustat. (D) RNA extracted from tissue slices presented in (B) and (C), reflecting the absence of toxicity of the drugs after 72 h of culture (normalized to the mean of untreated slices). Mean ± SEM; *p < 0.05, **p < 0.01, ***p < 0.001; Mann-Whitney test.

We infected OCCs with a recombinant MeV strain expressing EGFP as a reporter and carrying SSPE-derived substitutions in the fusion protein (S103I; N462S; N465S) that make virus entry and cell-to-cell propagation receptor-independent [8]. OCCs were treated with PHD inhibitors (*i.e.* Molidustat, Roxadustat or Daprodustat) 1 h after infection. Since MeV propagates relatively slowly in tissue slices, we considered pretreatment unnecessary. Viral growth was assessed by fluorescence microscopy (Figure 5A) and by RT-qPCR (Figure 5B and C) at 72 h post-infection – a time point allowing several cycles of viral replication to be completed. As shown in Figure 5A, overall EGFP expression decreased in OCCs treated with Molidustat or Roxadustat. Quantification of viral RNA by RT-qPCR in the first series of experiments confirmed the significant inhibition of MeV replication by Molidustat (Figure 5B). In the second series of experiments, we extended these findings to other PHD inhibitors, including Daprodustat and Roxadustat, which showed similar antiviral effects (Figure 5C). The fact that these three PHD inhibitors, which are chemically unrelated, have the same effect indicated that induction of the HIF pathway was responsible for the observed inhibition of MeV infection. Concordant results were obtained with a hyperfusogenic MeV variant carrying a different F protein substitution (Q96P; Supplementary Fig. 5) [9]. Total RNA levels in OCCs remained stable across all treatment conditions (*i.e.* Molidustat, Roxadustat or Daprodustat; Figure 5D), indicating that these drugs were not toxic during the 72-h culture period. We also demonstrated that PHD inhibitors did not affect the viability of OCCs as assessed by MTT assay (Supplementary Fig. 6). Together, these findings establish that the induction of the HIF pathway via PHD inhibition confers significant protection against MeV infection in a relevant brain tissue model, without compromising tissue viability.

**Figure 6.**
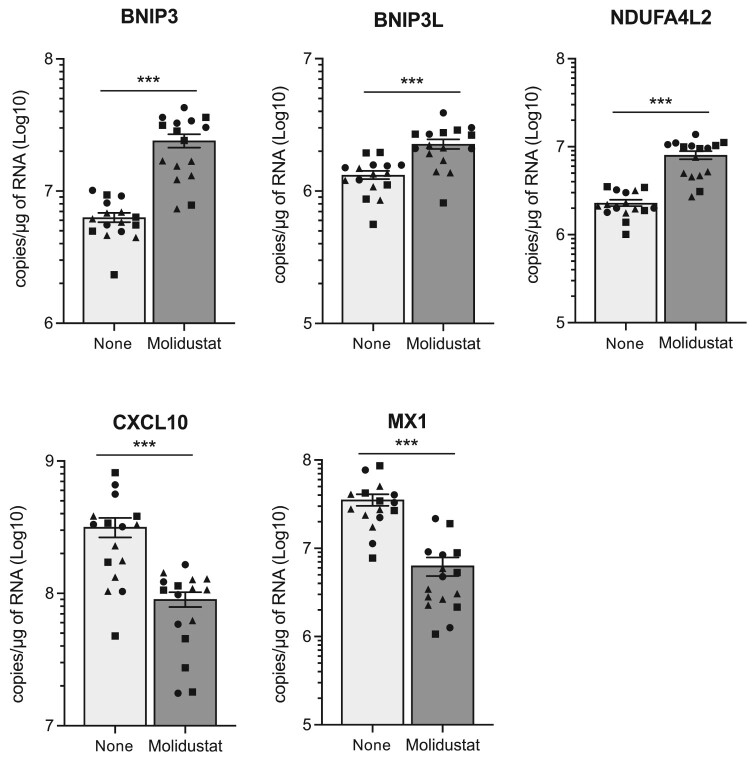
Molidustat induces hypoxia response genes but inhibits CXCL10 and Mx1 expression in organotypic brain cultures infected with MeV. Hamster OCCs were infected with hyperfusogenic MeV-EGFP (carrying S103I, N462S and N465S mutations in the F protein; 10,000 PFU/slice) and one later, cultures were treated with DMSO alone or Molidustat at 25 µM. After 72 h of culture, total RNA was extracted and the expression of BNIP3, BNIP3L, NDUFA4L2, CXCL10 and Mx1 was measured by RT-qPCR. Each dot represents one tissue slice from one animal. The whole experiment has been repeated three times, and replicates are identified by closed circles, squares and triangles. Mean ± SEM; ***p < 0.001; Mann-Whitney test.

We investigated whether this antiviral effect in OCCs could be related to the induction of interferon-related or inflammatory factors. GSEA analysis showed a significant enrichment for MSigDB genes associated with the “inflammatory” response, but only 4.2% mapped to the “Inflammation” gene set, and key inflammatory cytokines such as CXCL8, IL-6 or IL-1 were not induced in OCCs (Supplementary Fig. 1B, 2A and B). Thus, the subset of inflammation-related genes induced by Molidustat in OCCs does not correspond to a typical inflammatory response. In addition, hallmark genes of the “interferon alpha” response set were not induced but repressed (Supplementary Fig. 1B, 2A, and 2B). This corroborates previous studies showing that HIF activation is often associated with suppression of the interferon-mediated antiviral response, although this could be context-dependent [35,36]. Finally, we established that in MeV-infected OCCs, Molidustat treatment decreased the expression of CXCL10 and Mx1, two marker genes of the innate antiviral response (Figure 6; lower panel). In contrast, BNIP3, BNIP3L and NDUFA4L2, three markers of HIF pathway activation, were induced as expected (Figure 6; upper panel). In conclusion, these results in OCCs further suggest that MeV restriction by PHD inhibitors is independent of the induction of a classical innate antiviral response.

### Molidustat inhibits NiV infection ex vivo

**Figure 7.**
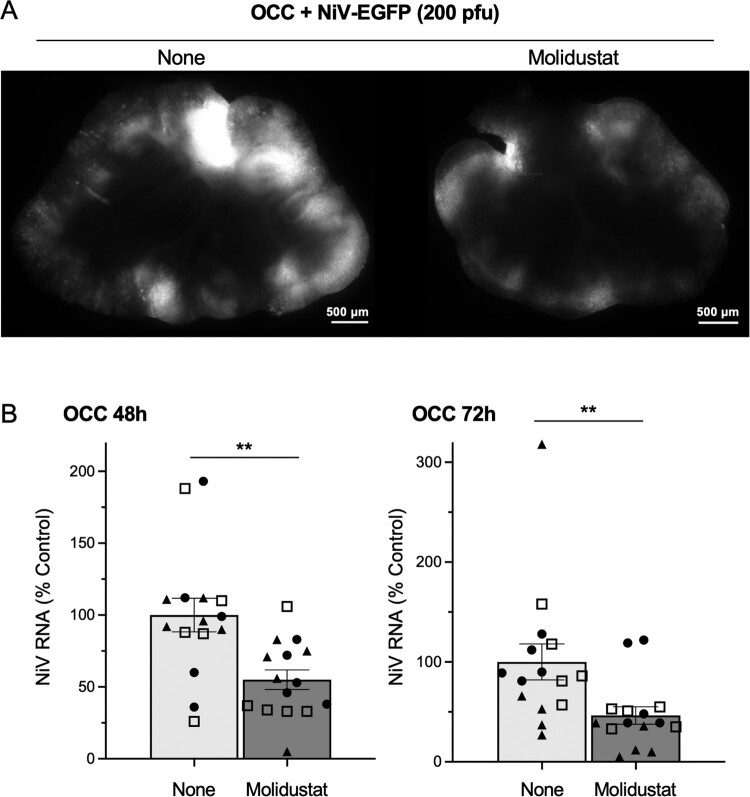
Pharmacological induction of the HIF pathway inhibits NiV replication in hamster organotypic brain cultures. (A) Hamster OCCs were infected with 200 PFU/slice of rNiV-EGFP. One hour after infection, Molidustat was added at 25 µM and NiV infection was analyzed after 48 h of culture by fluorescence imaging. (B) Quantification of NiV infection in OCCs after 48 h or 72 h of culture by RT-qPCR. Each dot represents one tissue slice from one animal. Data correspond to the results of three independent experiments, and replicates are identified by open squares, closed circles, and closed triangles. For each tissue slice, NiV-N RNA levels were first normalized to hamster RPL13A mRNA level, then to the average signal obtained across all DMSO-treated slices of the corresponding experiment. Mean ± SEM; **p < 0.01; Mann-Whitney test.

Since NiV belongs to the *Henipavirus* genus, which is closely related to the *Morbillivirus* genus, and has previously been shown to infect hamster OCCs and organotypic lung cultures (OLCs) [17], we tested the antiviral effect of Molidustat in OCCs and OLCs infected with this BSL4 pathogen. First, OCCs were infected with a recombinant NiV strain expressing EGFP and immediately treated with Molidustat or a vehicle control (DMSO). Fluorescence imaging showed efficient infection by NiV in DMSO-treated OCCs and a marked decrease of EGFP expression in Molidustat-treated slices, suggesting an inhibitory effect on viral propagation (Figure 7A). Viral RNA quantification by RT-qPCR at 48 and 72 h post-infection confirmed that Molidustat significantly inhibited NiV replication (Figure 7B). To determine whether Molidustat could exhibit antiviral effects beyond brain tissues, we further tested it in hamster OLCs, which represent another tissue targeted by NiV. Molidustat did not affect the viability of OLCs as assessed by MTT assay (Supplementary Fig. 6), and significantly inhibited NiV infection in these tissues (Supplementary Fig. 7). Therefore, we confirmed that its antiviral activity was not limited to the brain. Overall, these findings demonstrate that inducing the hypoxia response pathway with Molidustat can restrict NiV infection in *ex vivo* models of brain and lung tissues.

## Discussion

In this study, we have shown that the activation of the HIF signalling pathway by PHD inhibitors can restrict MeV and NiV infection both *in vitro* and *ex vivo* in hamster brain and lung organotypic cultures. Given that a similar antiviral effect has been observed with Molidustat, Roxadustat and Daprodustat, three PHD inhibitors with different chemical structures, we can rule-out an off-target effect that could be associated with any of them and confirm the crucial role of PHD in *Paramyxovirus* infectious cycle. Besides, the inhibitory effect of Molidustat on MeV infection was abolished by knocking-down HIF-1/2 expression, demonstrating the critical role of HIF signalling in this antiviral activity. In contrast, the knock-down of ATG5 showed no effect, suggesting that canonical, ATG5-dependent autophagy is not involved, contrary to recent reports implicating this factor in the antiviral effect of HIF-inducing molecules on HSV-2 in the neuroblastoma cell line SH-Sy5y [31]. Because HIF is a master regulator, notably controlling many metabolic factors, the downstream events explaining its antiviral effect remain to be identified although we have ruled out the induction of classical interferon-related or inflammatory responses.

Viral infections and the hypoxia response are interconnected through multiple mechanisms [37]. First, tissue damage caused by the virus can directly trigger hypoxia, as evidenced by impaired gas exchange in infected lungs or ischemia following complement activation and thrombosis. Under physiological O_2_ levels, HIFs can also be induced by the activation of the immune response. For example, Toll-like receptor 4 (TLR4) signalling in dendritic cells leads to HIF-1 upregulation [38], driving metabolic reprogramming, modulating the expression of inflammatory cytokines and chemokines, and activating other defense mechanisms such as the production of reactive oxygen species [39]. Finally, several viruses such as Hepatitis E virus (HEV), Porcine Reproductive and Respiratory Syndrome virus (PRRSV) and SARS-CoV-2 have developed mechanisms to hijack the HIF pathway and promote a glycolytic switch that increases energy production and metabolite availability for viral replication [40–43]. However, as interactions between the virus and the host metabolism and immunity are dynamic and involve feedback loops, the consequences of HIF activation on virus replication, inflammation, tissue damage, and pathogenesis are complex. Although several studies have established that HIFs promote viral replication [41,44–53], others have reported antiviral roles for these transcription factors [30,31,54–58]. Besides, HIF-1α has been implicated in the cytokine storm associated with SARS-CoV-2 infection [42]. In contrast, the HIF pathway in neurons from HSV2-infected mice restricts viral replication and limits inflammation in the brain [31]. These complex, sometimes opposing effects are illustrated by studies on influenza A virus (IAV). Indeed, HIF-1α suppression in A549 lung epithelial cells was shown to impair [45,52,53] IAV replication, but one study reported opposite effects [55]. Besides, separate *in vivo* studies reported that pharmacological inhibition or knock-down of HIF-1α decrease IAV replication and lung inflammation [45,52], whereas complete deletion of HIF-1α aggravates inflammation and lung injury [55]. Pharmacological induction of HIFs by the PHD inhibitor ML228 was also reported to inhibit IAV replication in neural cells SH-Sy5y [31]. These divergent outcomes, depending on experimental settings, highlight the complexity of HIF-virus interactions. In the present study, we have also evaluated the effects of Molidustat on organotypic mouse lung cultures (mOLC) infected with IAV H3N2 strain and found no inhibition of IAV replication in the infected tissue (Supplementary Fig. 8). While MeV and NiV replicate in the cytosol, the nuclear replication of IAV may protect the virus from antiviral mechanisms induced upon activation of the HIF pathway. Differences in the response of mouse and hamster lung tissue to PHD inhibitors must also be taken into account. Further investigations aimed at better understand the mechanisms involved in the restriction of MeV and NiV by PHD inhibitors should help explain the absence of effect on IAV.

Despite the abundance of literature on the interplay between various viruses and the hypoxia response pathway, little is known about the role of HIFs in *Paramyxoviridae* infections. Conflicting results have been reported for human respiratory syncytial virus (RSV), the prototype virus of the *Pneumoviridae* family that is close to *Paramyxoviridae* in the *Mononegavirales* order. Some studies have shown that RSV stimulates the HIF-1α pathway, promoting glycolysis and RSV growth [49,50,59] while HIF-1α suppression with siRNA or with the pharmacological inhibitor PX-478 impairs RSV progeny production in HEp-2 cells without affecting other steps of the viral replication cycle [49,50]. In contrast, Zhuang X. et al. established that HIF-inducing drugs such as Molidustat, Daprodustat and Roxadustat can suppress RSV infection in several cell lines [58], with the strongest effect observed when the drugs are administered 24 h before infection, thus mirroring our observations with MeV. They have also shown the *in vivo* efficacy of Daprodustat treatment against RSV infection [58]. Taken together, these findings suggest that basal HIF expression may support viral replication, while drugs that either inhibit or increase HIF expression can inhibit it via distinct mechanisms, potentially involving innate immune activation, translation repression, or autophagy [30,31,55,60].

The ambivalent impact of HIFs on viral growth underlines the importance of using relevant models to properly assess the potential antiviral effect of HIF modulators. Here, we used not only *in vitro* infection models but also *ex vivo* organotypic cultures of brain and lung slices, which preserve the native tridimensional architecture and primary cell populations in the organ and were shown to be highly predictive for *in vivo* experiments [17]. In this setting, HIF-inducing drugs inhibit MeV and NiV infections. However, such *ex vivo* models have inherent limitations, including the absence of blood circulation, the artificial composition of the culture medium, the lack of blood–brain barrier, and an immune system limited to that present within the tissue. Therefore, future studies should aim first to validate our findings *in vivo* either by using such molecules as part of a combinatory treatment or after enhancing their antiviral efficacy through chemical modification. A key point for evaluating the antiviral effect of HIF-inducing drugs *in vivo* is their bioavailability and pharmacokinetic. Interestingly, Roxadustat has been shown to penetrate the brain in animal models [61], and showed benefits in the treatment of acute ischemic stroke and glioblastoma growth in an orthotopic tumour model [62,63]. Besides, administration of Roxadustat to humans at 100 mg achieves blood concentrations which are compatible with the antiviral activity we have observed *in vitro* and *ex vivo* [64]. Small-animal models of MeV and NiV infection will also be required, as access to non-human primates is possible but often limited for both ethical and economic reasons [1,65]. Unfortunately, MeV infection in rodents remains quite artificial, as it typically requires transgenic animals expressing the MeV receptor or the use of highly-adapted viral strains. In addition, intracranial virus injection is often required to study neuroinvasion. Ferrets infection with Canine Distemper Virus (CDV), a morbillivirus closely related to MeV, is an interesting alternative to evaluate antivirals although it requires a specific animal facility [1]. Regarding NiV, the golden hamster is a relevant *in vivo* model for both viral replication and pathogenesis [65]. If testing is performed in MeV-infected ferrets or NiV-infected golden hamsters, the pharmacokinetic parameters of the candidate drugs will first need to be established for these animal models to ensure the success of this experiment.

Provided that antiviral effects are validated *in vivo*, repurposing HIF-inducing drugs against *Paramyxoviridae* infections is attractive, probably as part of a combinatory treatment. Indeed, these drugs have an excellent safety profile and despite legitimate concerns, particularly with regard to the risk of cancer development, cardiovascular events or thromboembolic complications, available clinical data are reassuring for short-term treatments [5]. Indeed, HIF-inducing drugs will only be administered for a few days to treat *Paramyxoviridae* infection, which will limit potential adverse effects. Finally, and given the critical clinical outcome of NiV or MeV infection of the brain, the benefit/risk ratio will be clearly in favour of the treatment. In conclusion, our results suggest that stimulating the HIF signalling pathway, as already achieved with drugs used in the treatment of chronic anemia, should be considered for treating acute severe infections caused by NiV and other *Paramyxoviridae*.

## Supplementary Material

SupplementaryFigure8R1.tiff

SupplementaryFigure3R1.tiff

SupplementaryTable1.xlsx

SupplementaryFigure5R1.tiff

SupplementaryFigure2R1.tiff

SupplementaryFigure7R1.tiff

SupplementaryFigure1R1.tiff

SupplementaryFigure4R1.tiff

SupplementaryFigure6R1.tiff
