# Supplementary figures and images for: In Vitro and Ex Vivo evidence that pharmacological induction of the hypoxia response pathway efficiently restricts measles and Nipah virus infections
Source: Emerg Microbes Infect. 2025 Sep 17;14(1):2563067. doi: 10.1080/22221751.2025.2563067 (PMC12536633; doi:10.1080/22221751.2025.2563067)

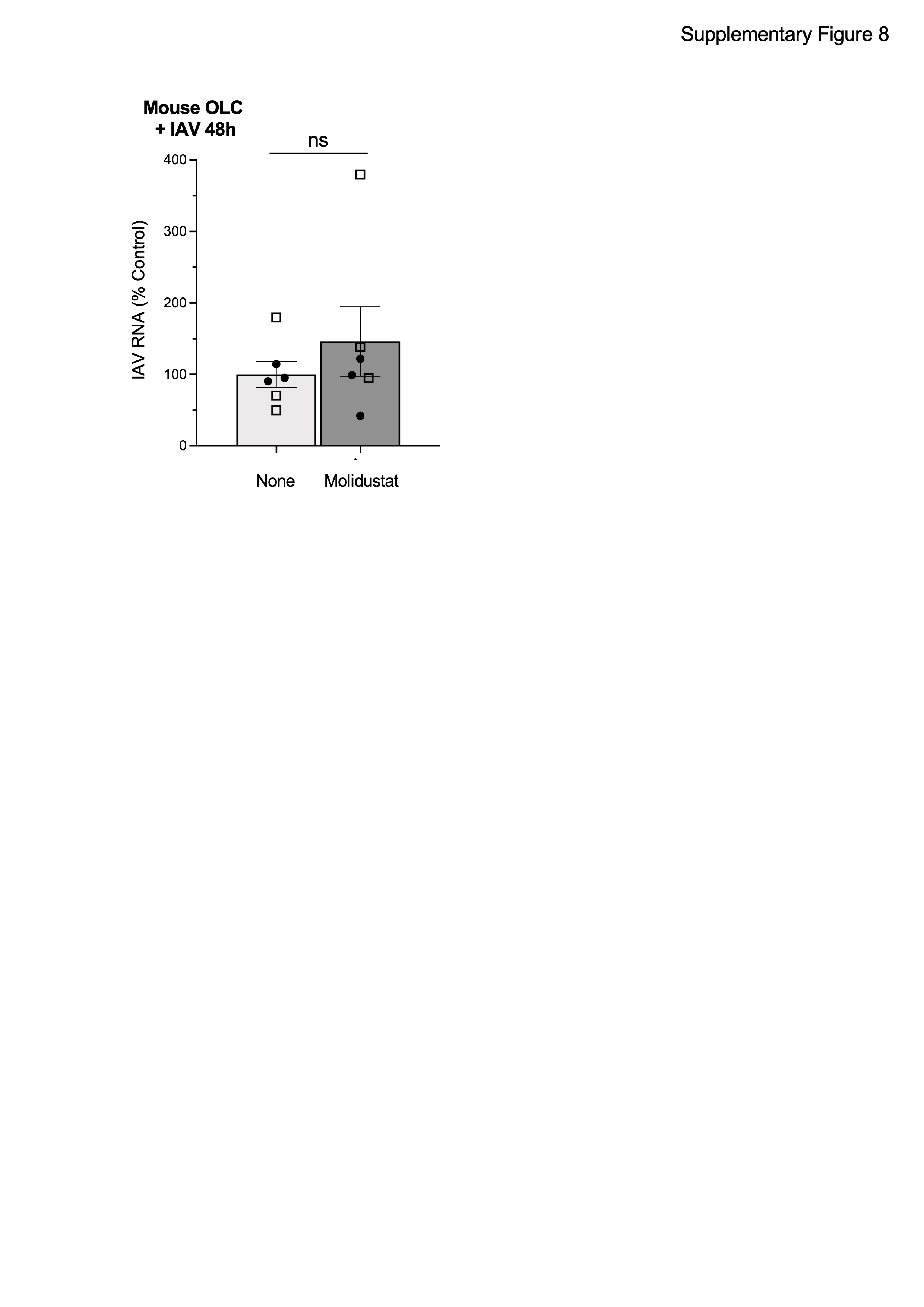

Supplement: SupplementaryFigure8R1.tiff [file TEMI_A_2563067_SM3257.tiff]

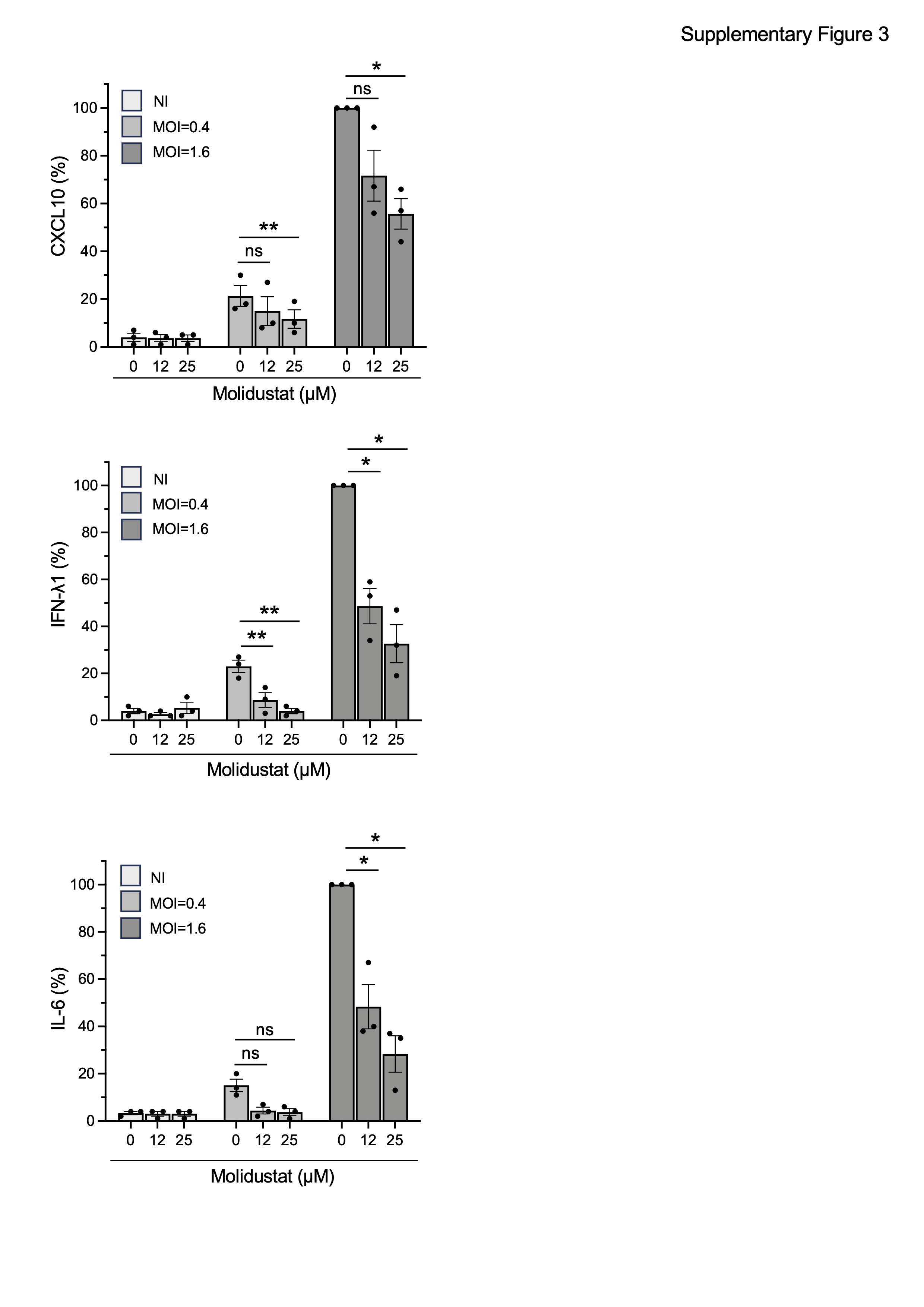

Supplement: SupplementaryFigure3R1.tiff [file TEMI_A_2563067_SM3256.tiff]

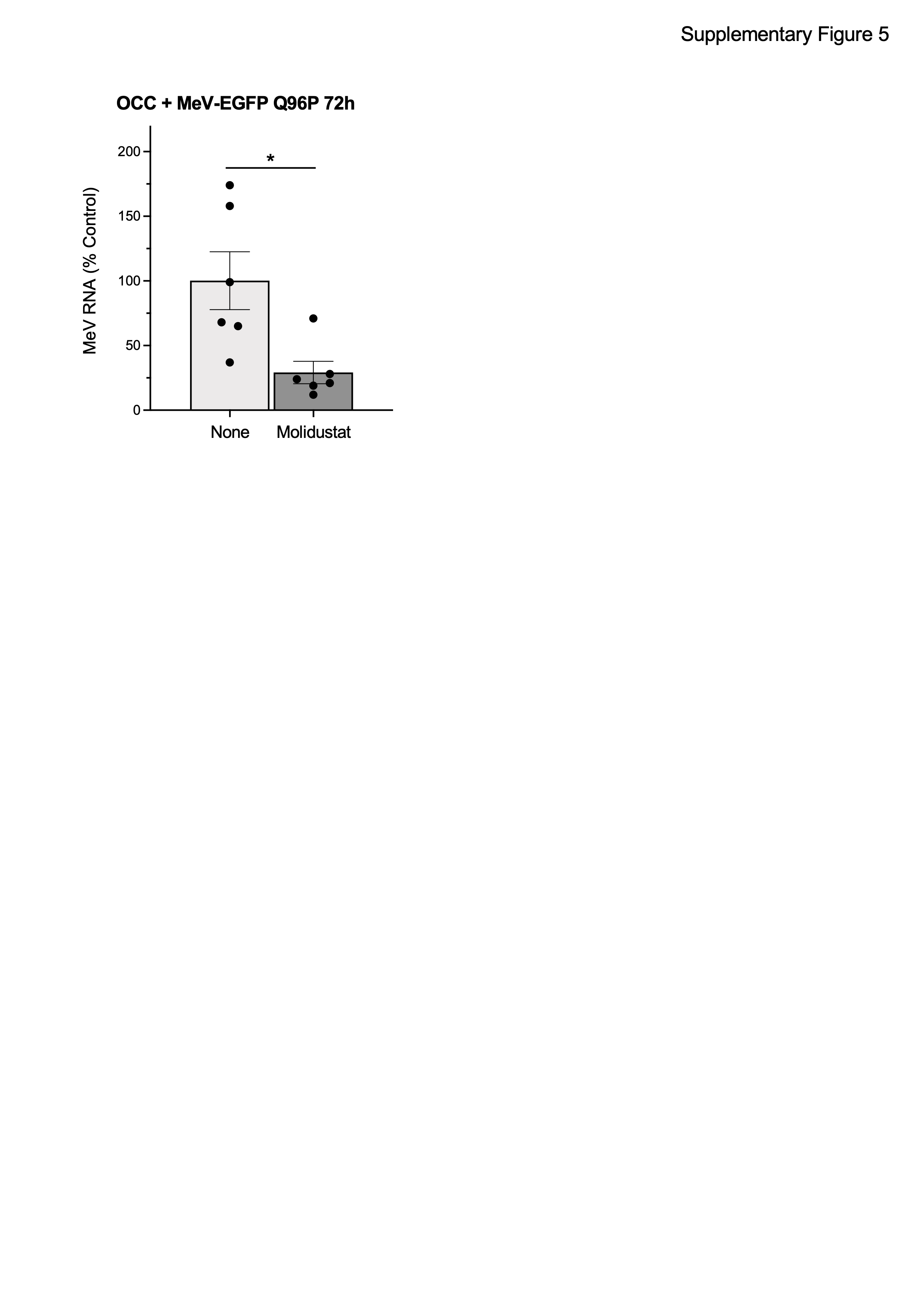

Supplement: SupplementaryFigure5R1.tiff [file TEMI_A_2563067_SM3254.tiff]

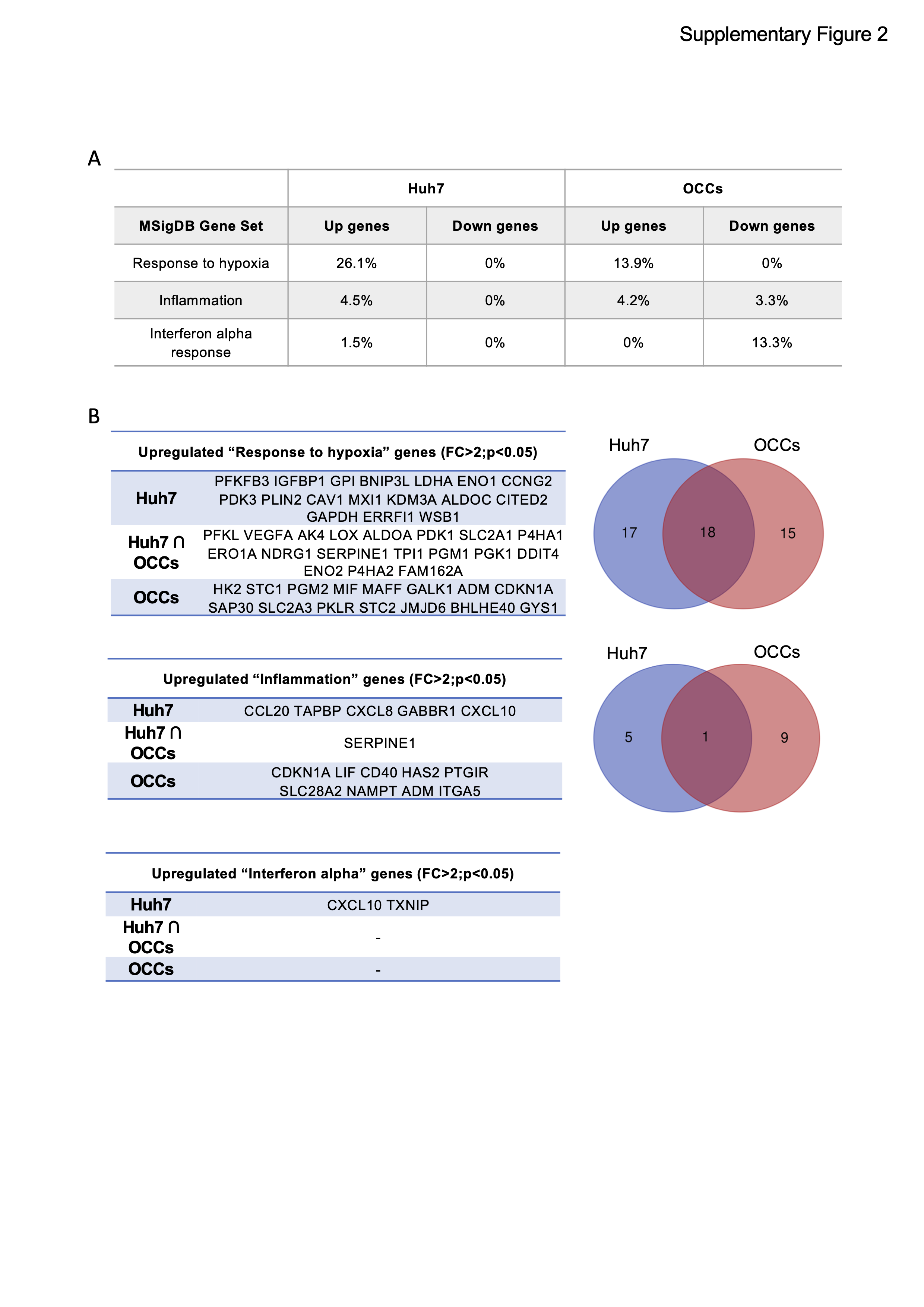

Supplement: SupplementaryFigure2R1.tiff [file TEMI_A_2563067_SM3253.tiff]

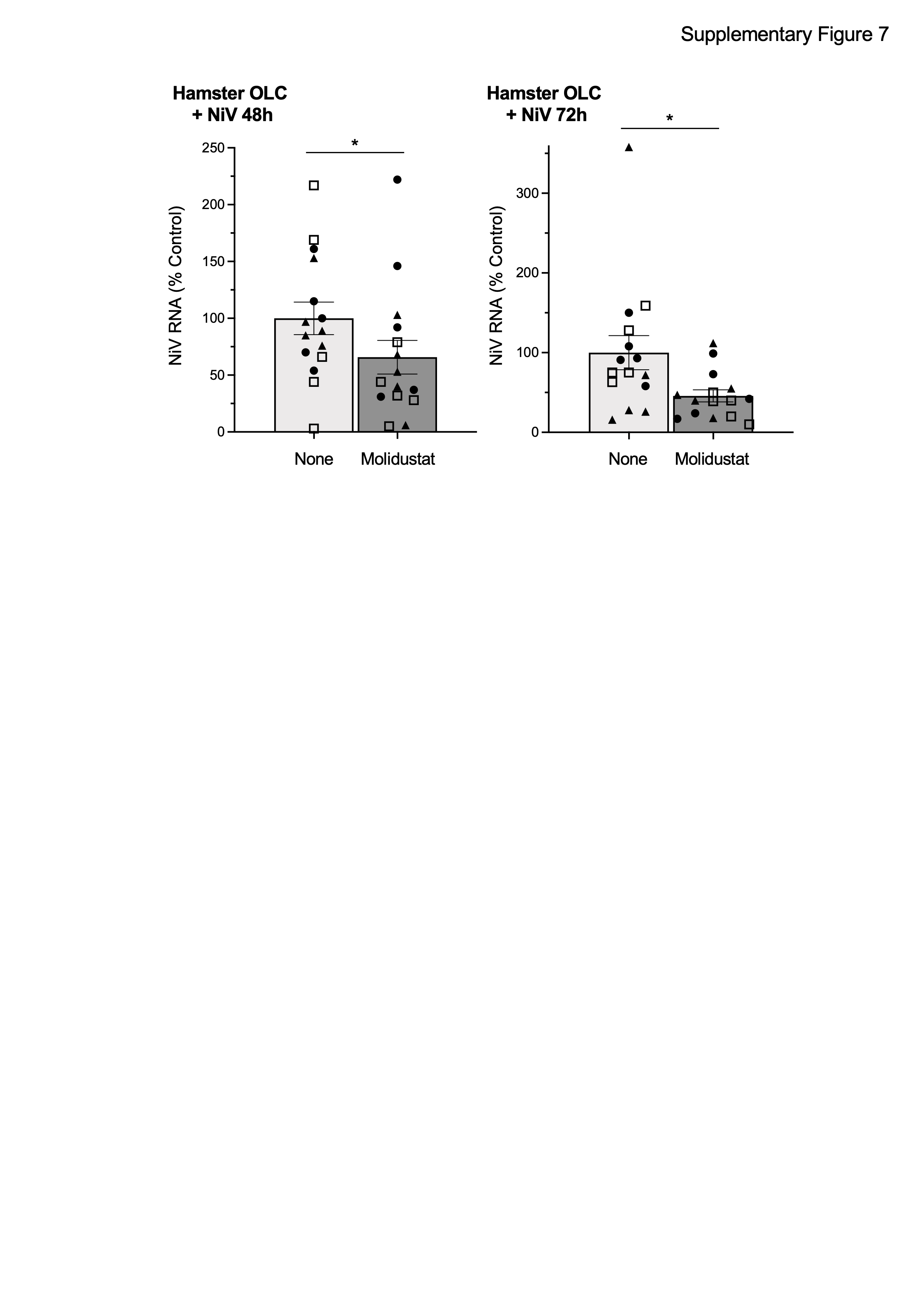

Supplement: SupplementaryFigure7R1.tiff [file TEMI_A_2563067_SM3252.tiff]

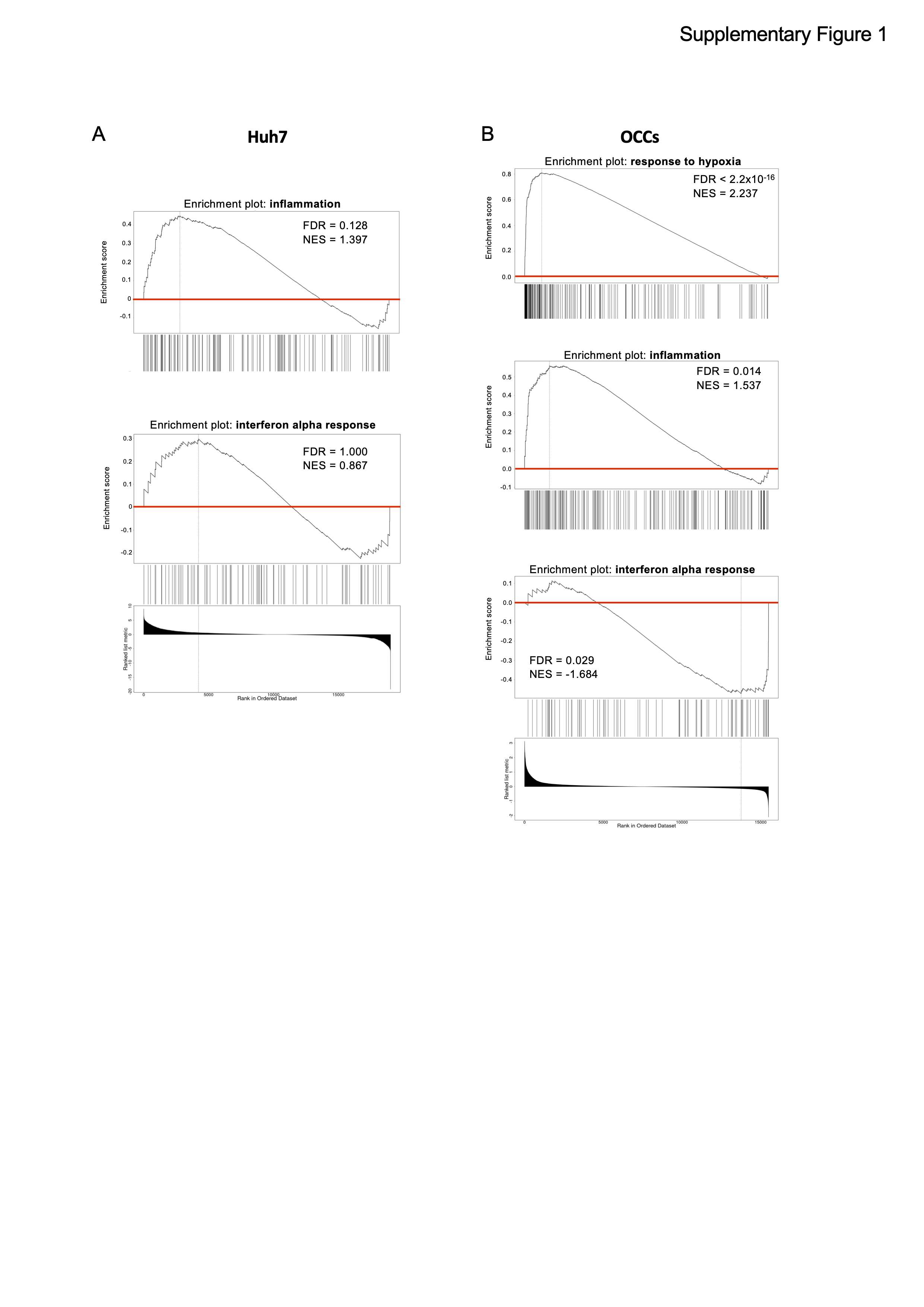

Supplement: SupplementaryFigure1R1.tiff [file TEMI_A_2563067_SM3251.tiff]

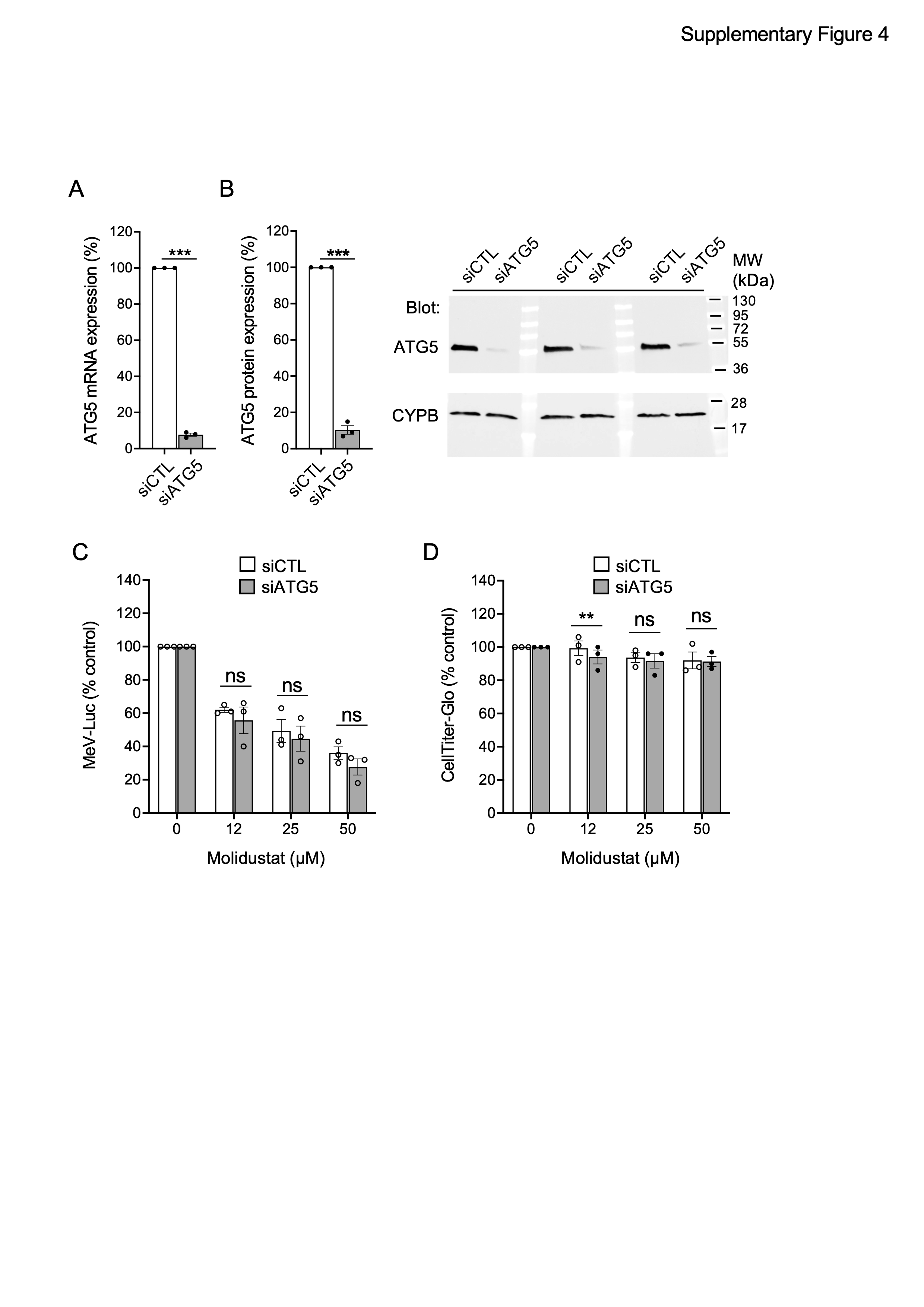

Supplement: SupplementaryFigure4R1.tiff [file TEMI_A_2563067_SM3250.tiff]

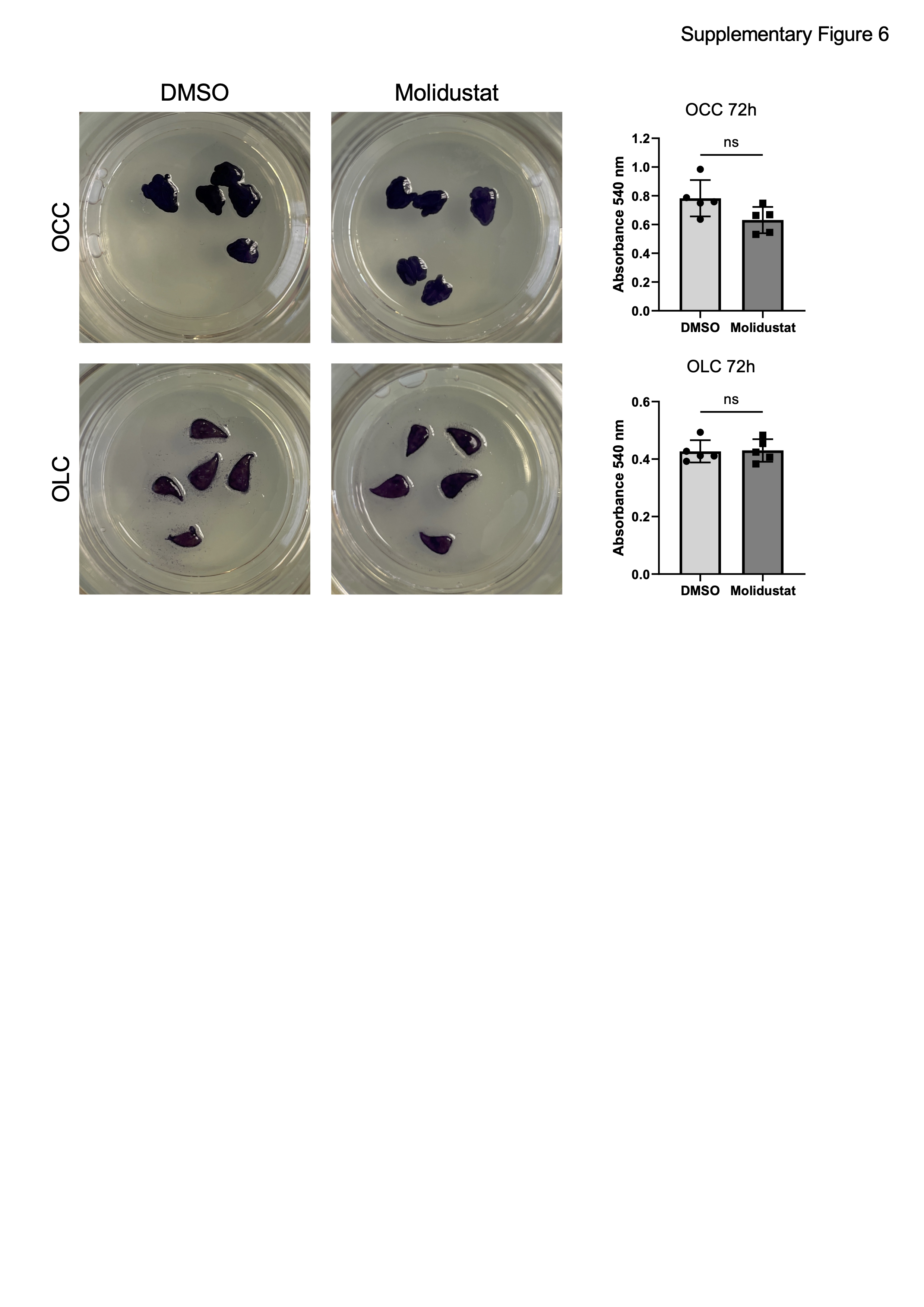

Supplement: SupplementaryFigure6R1.tiff [file TEMI_A_2563067_SM3249.tiff]
